# Synchronous Adenocarcinoma and Mucosa-associated Lymphoid Tissue Lymphoma of the Colon

**DOI:** 10.4103/1319-3767.74455

**Published:** 2011

**Authors:** Padmalaya Devi, Lucy Pattanayak, Sagarika Samantaray

**Affiliations:** Department of Surgical Oncology, A.H. Regional Cancer Centre, Cuttack, India; 1 Department of Radiation Oncology, A.H. Regional Cancer Centre, Cuttack, India; 2 Department of Oncopathology, A.H. Regional Cancer Centre, Cuttack, India

**Keywords:** Adenocarcinoma, MALT lymphoma, synchronous

## Abstract

Mucosa-associated lymphoid tissue (MALT) tumors are a distinct subtype of non-Hodgkin’s lymphoma. Synchronous appearance of adenocarcinoma and colonic MALT lymphoma in the same patient is quite rare. In the present report, we describe a 68-year-old female who presented with a history of bleeding per rectum. She had no history of fever, loss of weight or drenching night sweats. Rectal examination revealed no abnormality. Colonoscopy showed a large ulceroproliferative mass arising from the hepatic flexure, biopsy of which came out to be adenocarcinoma of colon. A right hemicolectomy was performed and microscopic study revealed the tumor type to be synchronous adenocarcinoma with lymphoma. The final diagnosis of this patient turned out to be a synchronous manifestation of both colonic adenocarcinoma and colonic MALT lymphoma. Although the patient remains asymptomatic two years after surgery, the case highlights the therapeutic dilemma that prevails in the definitive management in such scenarios.

Mucosa-associated lymphoid tissue (MALT) tumors are a distinct subtype of non-Hodgkin’s lymphoma associated with predisposing infectious or autoimmune process, resulting in chronic lymphoid proliferation. Though stomach is the most common site, MALT tumor has been reported in non-gastric sites like salivary gland, lung, ocular adnexa and skin.[1] MALT tumors affecting multiple organs (multiorgan MALT) have been reported in literature. Although large intestinal MALT lymphomas are rare but majority of patients with multiorgan MALT tumor have preferential involvement of large intestine.[2] Another rare presentation is simultaneous occurrence of MALT tumor with a carcinoma. The synchronous appearance of both colonic adenocarcinoma and colonic MALT lymphoma in the same patient is a previously unknown entity. In the present report, we describe a case of synchronous colonic carcinoma and MALT lymphoma.

## CASE REPORT

**Figure 1:**
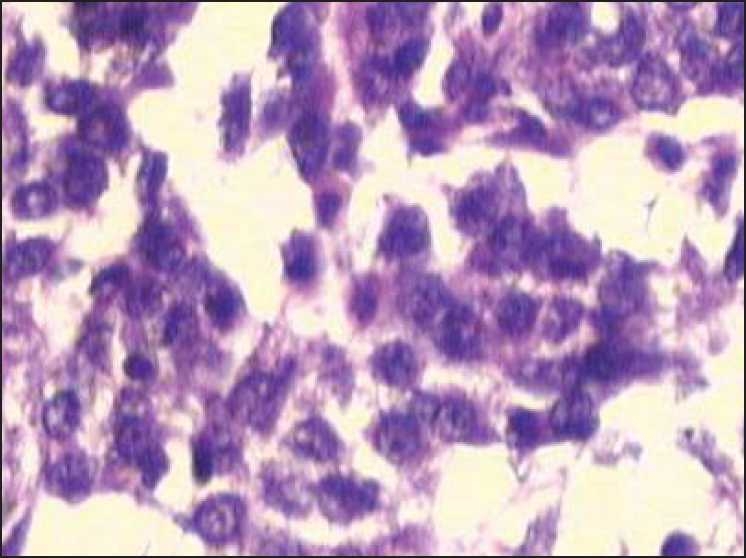
Malignant looking tumor cells arranged in clusters and acinar pattern, containing mucin suggestive of adenocarcinoma

**Figure 2:**
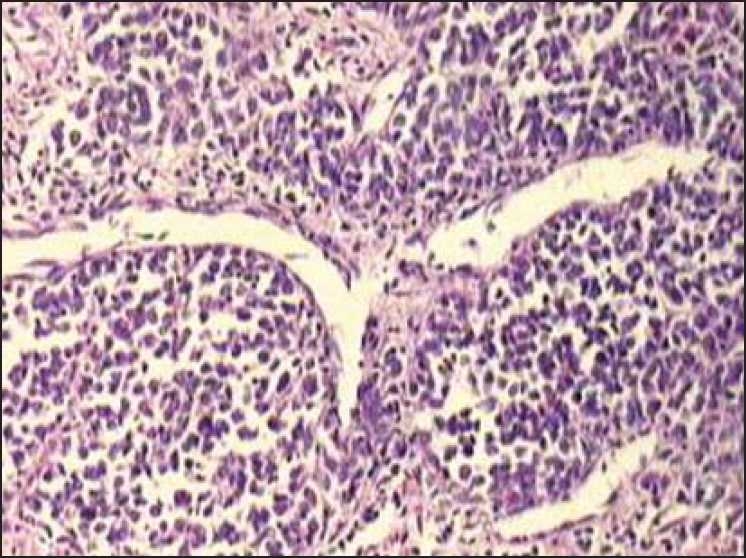
Malignant looking lymphoid cells closely adherent and dispersed along a blood vessel suggesting angiocentricity as seen in high grade lymphomas. Also seen are cells arranged in clusters or acinar pattern suggestive of adenocarcinoma

**Figure 3:**
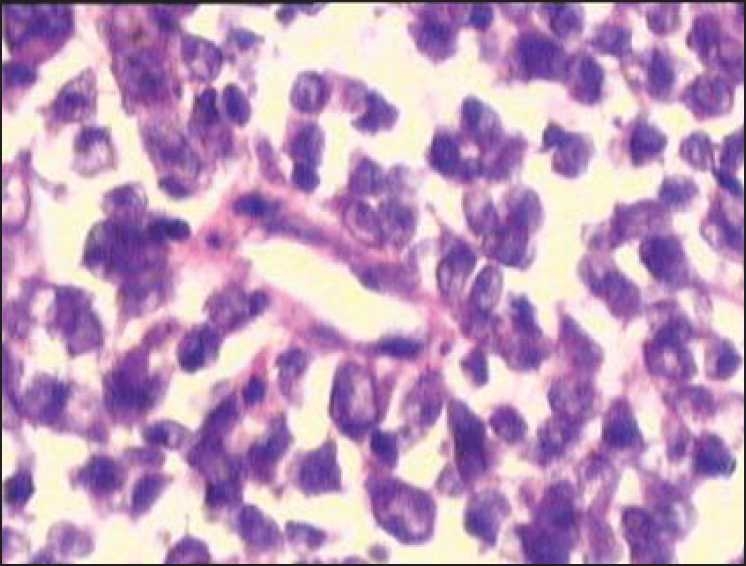
Infiltration of malignant looking lymphoid cells in the lamina propria suggestive of malt lymphoma

**Figure 4:**
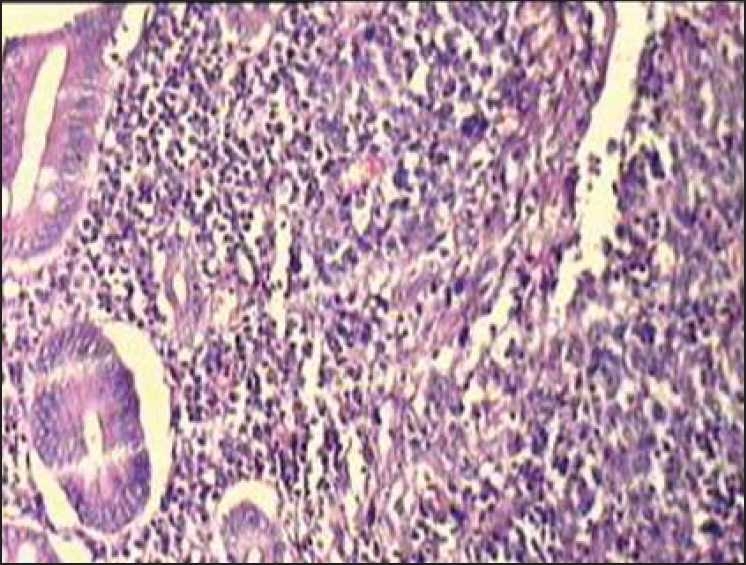
Small to medium sized lymphoid cells having scant to moderate pale eosinophilic cytoplasm. Nucleus is grooved or cleaved with prominent nucleoli, vesicular chromatin. Cells are dispersed along the blood vessel showing angiocentricity and compressing the blood vessel into a slit like appearance

A 68 year old female presented to the surgical clinic with history of bleeding per rectum. Her medical and family history was not significant. She had no history of fever, loss of weight or drenching night sweats. On physical examination, she was poorly built, severely pale and nonicteric. There was no peripheral lymphadenopathy or hepatosplenomegaly. There were no abnormal findings on rectal examination. Colonoscopy revealed a large ulceroproliferative mass arising from the hepatic flexure of size 4×3 cms. The biopsy of the lesion revealed adenocarcinoma of colon. Chest radiography revealed normal architecture of the thorax and lung parenchyma. The abdominal sonography revealed a large hypoechoic mass of size 6 ×4 cms arising from the hepatic flexure of colon, with no evidence of liver metastases or abdominal lymphadenopathy. On laparotomy, a huge, mobile, ulceroproliferative growth was seen arising from the hepatic flexure, not infiltrating the surrounding structures. No suspicious lymphadenopathy or metastases were seen in the liver, peritoneum. A right hemicolectomy was performed. The gross pathological examination of the lesion showed a 6×4×3cm, cauliflower like proliferative tumor present in the mucosal surface. Entire mucosal surface appeared normal without any abnormality or polypoidal lesion. Three small lymph nodes varying in size from 0.5 to 0.8 cm were isolated from pericolic fat. The microscopic study revealed the tumor type to be synchronous adenocarcinoma with lymphoma. The adenocarcinoma component was moderately differentiated (grade 2) while the lymphoma component was of high grade MALT lymphoma variety [Figures 1 and 2]. The lymphomatous component showed evidence of local invasion into the subserosa [Figures 3 and 4]. The surgical cut margins were free of tumor and all three lymph nodes isolated from the pericolic fat lacked tumor dissemination. Two months after surgery, the patient remained asymptomatic.

## DISCUSSION

Second malignancies are classified as synchronous or metachronous. According to Gluckman’s definition[3] “synchronous carcinomas” include carcinomas that present either simultaneously or within a six-month period of identification of the original tumor. Carcinomas diagnosed beyond the 6-month interval are referred to as “metachronous carcinoma”. Our case fits the definition of synchronous malignancies.

Gastrointestinal tract is the most frequently involved extra-nodal site in non-Hodgkin’s lymphoma, stomach being the most common (50-60%) followed by small intestine (30%).[4] Several case details of colonic MALT lymphoma have been reported in literature but the present case of synchronous colonic MALT lymphoma and carcinoma in the same colonic segment is the first case of its kind to be reported, to the best of our knowledge. Moriya *et al*.[5] reported a case in which diagnosis of synchronous malignant lymphoma was made on 2^nd^ surgery, a month after patient was diagnosed with colonic adenocarcinoma after right hemicolectomy. Another case of adenocarcinoma cecum with simultaneous follicular lymphoma of the terminal ileum and regional nodes was reported by Bhanote *et al*.[6]

Though several risk factors associated with development of colonic carcinoma are known, the role of causative factors in MALT lymphoma genesis is not well established. Various infectious agents like *H. pylori*, hepatitis C virus have been implicated as a possible link in the development of non-gastric MALT. Because of the extreme rarity of this scenario, limited information is available to decide on any standard treatment. Surgery is considered to be the mainstay of treatment and the role of radiotherapy and type of chemotherapy remains unclear. Additional adjuvant treatment was not given in this case, because of patient’s refusal.

## CONCLUSION

We investigated a rare case of synchronous malignancy (adenocarcinoma and MALT lymphoma) in colonic region. A therapeutic dilemma exists in deciding the course of treatment in patients with synchronous malignancies. More cases should be reported in literature so as to formulate a definite line of management in such difficult scenarios.
